# Electrically Tunable and Negative Schottky Barriers in Multi-layered Graphene/MoS_2_ Heterostructured Transistors

**DOI:** 10.1038/srep13743

**Published:** 2015-09-03

**Authors:** Dongri Qiu, Eun Kyu Kim

**Affiliations:** 1 Quantum-Function Research Laboratory and Department of Physics, Hanyang University, Seoul 133-791, South Korea

## Abstract

We fabricated multi-layered graphene/MoS_2_ heterostructured devices by positioning mechanically exfoliated bulk graphite and single-crystalline 2H-MoS_2_ onto Au metal pads on a SiO_2_/Si substrate *via* a contamination-free dry transfer technique. We also studied the electrical transport properties of Au/MoS_2_ junction devices for systematic comparison. A previous work has demonstrated the existence of a positive Schottky barrier height (SBH) in the metal/MoS_2_ system. However, analysis of the SBH indicates that the contacts of the multi-layered graphene/MoS_2_ have tunable negative barriers in the range of 300 to −46 meV as a function of gate voltage. It is hypothesized that this tunable SBH is responsible for the modulation of the work function of the thick graphene in these devices. Despite the large number of graphene layers, it is possible to form ohmic contacts, which will provide new opportunities for the engineering of highly efficient contacts in flexible electronics and photonics.

## Introduction

Among layered 2-dimensional (2D) materials, molybdenum disulfide (MoS_2_) is attracting attention as a semiconducting material in the transition metal dichalcogenide family^1^,^2^. Because of quantum mechanical confinement, MoS_2_ possesses several remarkable properties, such as an absence of dangling bonds^3^, a lack of inversion symmetry^4^, valley degrees of freedom^5^, and other fascinating physical properties; as a result, it shows promise as a novel candidate material for high-performance and low-power electronics applications^6^. Moreover, the individual layers, which interact through the van der Waals force, can be readily exfoliated into atomically thin layers^7^, and when this method is applied to combine MoS_2_ with other 2D materials, novel heterostructured devices can be constructed^8^. Examples of these types of structures that have been reported in the literature include vertical tunneling transistors^9^,^10^, hybrid graphene/MoS_2_ photoresponsive devices^11^, and memory devices that incorporate hexagonal boron nitride^12^. Although multifunctional device architectures offer various promising functionalities, various aspects of the performance-degradation effect have not been fully explored. In particular, previous studies have shown that the metal/MoS_2_ and gate dielectric/MoS_2_ interfaces significantly affect device performance^13^,^14^,^15^. In the case of the metal/MoS_2_ interface, there is a scalable Schottky barrier height (SBH) that restricts carrier injection when a very low-work-function metal, such as scandium, is used^13^. Several previous works on contact optimization using single-layer graphene have been affected by the same difficulties^16^,^17^. However, very recently, Yu, L. *et al.*^18^ and Liu, Y. *et al.*^19^ have observed a barrier-free contact by using single-layer graphene as the back interconnection. Nevertheless, ohmic contact has been rarely achieved with relatively thick graphene (or graphite), and few detailed studies on the back-gate tunability of negative barrier heights have been conducted.

In this study, we present heterostructured MoS_2_ field-effect transistors (FETs) that contain approximately 20 layers of graphene (denoted below as MGr) to form bottom-up interconnections for source and drain electrodes. Here, an alternative approach was engineered by inserting a thick graphene layer instead of single-layer graphene. To compare the characteristics of the devices, reference samples without MGr contacts were also fabricated. The heterostructured FETs exhibited a dramatic reduction in SBH based on the framework of the thermionic emission theory. It appeared that the observed negative SBH gave rise to true ohmic contact.

## Results

### Fabrication of the devices

**Figure 1:**
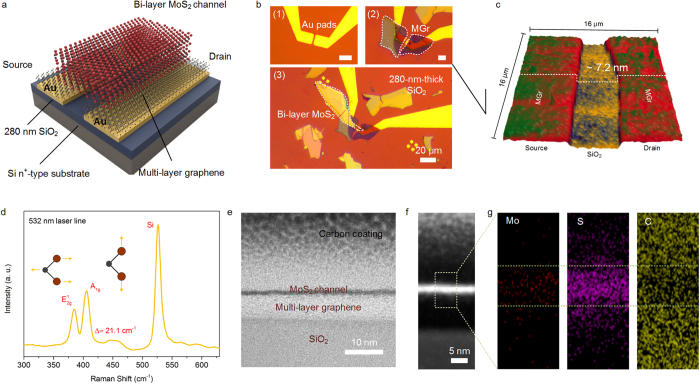
Fabrication process and schematic illustration of an MGr/MoS_2_ device. (**a**) A 3D schematic image of our back-gated MoS_2_ FET. (**b**) An optical image of pre-patterned Au pads (1), MGr flakes deposited on top of the Au electrodes (2), and the separation distance between them, which is defined as the channel length (L ~ 3.5 μm), as well as an optical image of the transfer of MoS_2_ flakes onto the top surface of the MGr/Au stack to form a semiconducting channel with a width of W ~5.2 μm (3). (**c**) 3D AFM topographic image of MGr source/drain electrodes deposited on a 280-nm-thick SiO_2_/Si substrate. (**d**) The Raman signal collected from the deposited MoS_2_ channel in the MGr/MoS_2_ FET and compared with that of MoS_2_ flakes, as shown in Figure S2. (**e**) Cross-sectional HRTEM image of the heterostructured FET. (**f**) STEM image showing the interface between the MGr and the few-layered MoS_2_ as well as the top of the carbon-Pt coating. (**g**) EDX mapping of the selected area for the Mo, S, and C elements.

A 3-dimensional (3D) schematic representation of our heterostructured device architecture is depicted in Fig. 1a. To investigate the electronic properties of the back-gated MGr/MoS_2_ heterostructured devices, we deposited mechanically exfoliated graphite and single-crystalline 2H-MoS_2_ onto a SiO_2_/Si substrate on which Au metal pads (110 nm thick) had been pre-patterned, as described in the Methods section. The dry transfer technique used for both 2D materials began with the introduction of an intermediate polydimethylsiloxane (PDMS) layer, which acted as a contamination-free viscoelastic material to avoid wet chemistry (more information can be found in the Supplementary Information, Figure S1). Two multi-layered graphene samples were transferred from the PDMS layer to serve as the source and drain, as shown in Fig. 1b. Subsequently, a MoS_2_ flake was directly transferred on top of the graphene flakes, overlapping both graphene contacts, to form a semiconducting channel using the same method (see (3) in Fig. 1b). For comparison, we also fabricated un-encapsulated bi- to quad-layer MoS_2_ FETs (denoted below as Au/MoS_2_) without inserting an MGr layer; for these devices, the transfer procedure was reduced to only one step. The MoS_2_ channel was verified using Raman spectroscopy and atomic force microscopy (AFM) to confirm the thickness of the MoS_2_ flakes (see Fig. 1d and the Supplementary Information, Figures S2 and S3). The frequency difference of 21.1 cm^−1^ between the in-plane mode (E^1^_2g_) and the out-of-plane mode (A_1g_) corresponds to the signal of bi-layer MoS_2_, which is consistent with our MoS_2_ reference sample (Figure S2) and other reports^20^. Optical microscopy and AFM were used to determine the MGr thicknesses, which were 7.1 and 7.3 nm for the source and drain, respectively, as shown in Fig. 1c. The average height of the graphene flakes was further characterized using statistical studies and was found to be ~7 nm (Figure S4). Cross-sectional high-resolution transmission electron microscope (HRTEM) analysis was used to study the overlapped MGr/MoS_2_ stack. An HRTEM image of a clear and compact interface, without other impurities or significant gaps between the MoS_2_ and MGr, is shown in Fig. 1e. Figure 1f displays a scanning TEM (STEM) image of the MGr/MoS_2_ heterostructure, and the corresponding energy-dispersive X-ray spectroscopy (EDX) mapping is shown in Fig. 1g and S5.

### Characterization of the devices

**Figure 2:**
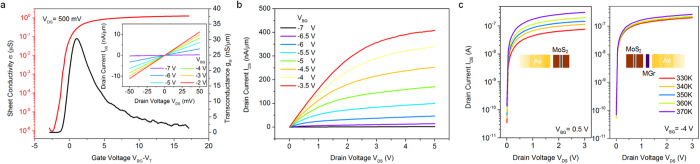
Electrical performance of the transistors. (**a**) Normalized I-V transfer characteristics of a typical back-gated MGr/MoS_2_ device at a fixed drain voltage. Inset: I_DS_-V_DS_ curve at a low drain bias (V = ±50 mV). The linearity was maintained under various gate voltages. (**b**) Output characteristics at various gate voltages. (**c**) Left: Temperature-dependent (from 330 to 370 K in 10-K increments) I_DS_-V_DS_ characteristics for an Au/MoS_2_ FET (left) and an MGr/MoS_2_ FET (right). The inset shows the corresponding device configurations.

To investigate the presence of the intermediate MGr layer in our MGr/MoS_2_ devices and its influence on the Schottky barrier, the devices were electrically characterized at room temperature. Figure 2a shows the typical I_DS_-V_BG_ transfer characteristics of the back-gated heterostructured FETs, which are presented as a normalized sheet conductivity (defined as σ = I_DS_/V_DS_(L/W)) as a function of V_BG_-V_T_, where V_BG_ is the gate voltage and V_T_ is the threshold voltage. The devices displayed obvious n-type behavior and produced a high on/off ratio (~10^6^) with a maximum normalized transconductance g_M_/W of approximately 30.4 nS/μm at V_DS_ = 500 mV. I-V hysteresis behavior is also shown in Figure S6. After *in situ* thermal heating, the devices exhibited nearly “hysteresis-free” behavior, indicating that the amount of adsorbates was negligible^21^. By contrast, the devices without an MGr layer demonstrated a transconductance of 0.35 nS/μm at V_DS_ = 500 mV, which is 2 orders of magnitude lower than that of the MGr/MoS_2_ FETs, as shown in Figure S7. The field-effect mobility, μ_FE_, was estimated from the maximum value of the transconductance using the expression μ_FE_ = g_M_L/WC_OX_V_DS_. Here, C_OX_ = ε_r_ε_0_/d_OX_ ~ 1.23 × 10^−8^ F/cm^2^ (ε_0_ = 8.854 × 10^14^ F/m, d_OX_ = 280 nm), which is the back-gate capacitance per unit area, and g_M_ = dI_DS_/dV_BG_ is the transconductance. Thus, the field-effect mobility for our bi-layer MGr/MoS_2_ device appeared to be approximately 17.9 cm^2^/Vs in the bi-layer MoS_2_ channel, as presented in Figure S8. This measured mobility is comparable to those of mono- or bi-layer MoS_2_ in high-κ gate dielectric capping devices^22^,^23^. The reference sample (thick bi-layer MoS_2_) with an Au metal contact had a mobility of 0.2 cm^2^/Vs, which is consistent with other reports^3^,^24^,^25^. Such a low mobility may be originated from the presence of a sizable Schottky barrier at the Au-MoS_2_ interface that hinders efficient carrier injection. To extract an accurate gate capacitance for the mobility calculation, a 100-kHz capacitance-voltage (C-V) profile was obtained by measuring between the source and back-gate electrodes (see Figure S9). The floor value of the capacitance (the minimum capacitance in the inversion regime) represented the actual electrode capacitance^26^, and the measured gate capacitance (~1.24 × 10^−8^ F/cm^2^) was very close to the value of 1.23 × 10^−8^ F/cm^2^ obtained in the above calculation for a 4.52-mm^2^ contact area.

### Extraction of the Schottky barrier height

Figure 2b displays the I_DS_-V_DS_ output characteristics of the MGr/MoS_2_ devices at various gate voltages. The saturation current could be reached at high drain voltages under certain gate biases, opening a wide drain voltage window. Moreover, the drain current, I_DS_, exhibited symmetrical and linear behavior (inset of Fig. 2a) under a small source-drain bias sweep (±50 mV). Previous studies have claimed that the linear relationship in the range of low V_DS_ bias suggests ohmic contact at the metal-to-semiconductor junction^27^. However, the present experimental findings imply the existence of a sizable energy barrier at the two back Schottky diodes based on detailed variable-temperature measurements. Remarkably, the I_DS_-V_DS_ characteristics at a fixed gate bias, on the right-hand side of Fig. 2c, manifested an extremely weak temperature dependence in the temperature range from 330 to 370 K compared with the Au/MoS_2_ sample (left-hand side of Fig. 2c). This weak temperature dependence implies a reduction in the barrier height for thermal emission due to the modification of the barrier at the MGr/MoS_2_ interface.

**Figure 3:**
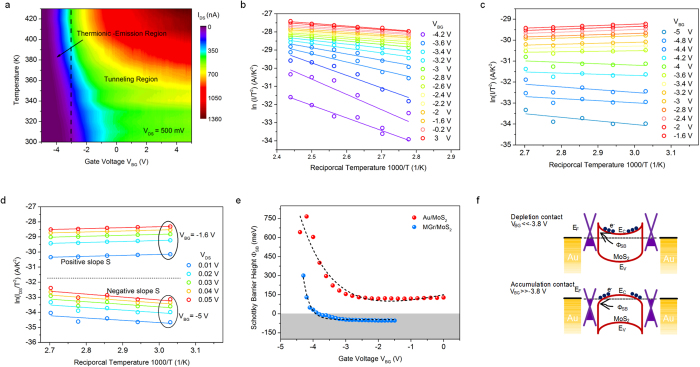
Temperature dependence of electrical transport and the extraction of the Schottky barrier height. (**a**) Color map of the temperature-dependent transfer characteristics at V_DS_ = 500 mV. (**b,c**) Arrhenius plots of ln(I_DS_/T^2^) *vs.* 1000/T at various gate voltages for an Au/MoS_2_ FET (**b**) and an MGr/MoS_2_ FET (**c**,**d**) Arrhenius plot for V_BG_ = −1.6 V and −5 V at various drain voltages from 10 to 50 mV. (**d**) Gate bias dependence of the variations in the Schottky barrier height. (**e**) Top: Schematic band diagram for a depletion-type contact. Bottom: Illustration of an accumulation contact.

To better understand how the carriers travel in the presence of a Schottky barrier, we employed a well-known model, *i.e.,* the 3D thermionic-emission theory, to describe our observations. Generally, the two carrier transport mechanisms are thermionic emission and quantum-mechanical tunneling under gate bias modulations, as shown in Fig. 3a. The former current mechanism is associated with the emission of majority carriers over the interfacial barrier, and the tunneling mechanism involves carriers crossing the barrier in a highly accumulated MoS_2_ channel. Under the assumption that the current is predominantly governed by the thermionic emission theory, the current I_DS_ is given by the expression^28^ I_DS_ = AA^*^T^2^exp(−eΦ_SB_/k_B_T)[exp(eV_DS_/k_B_T) − 1], where A^*^, A, e, V_DS_, and k_B_ are the Richardson constant, the area of the contact, the elemental charge, the drain voltage, and the Boltzmann constant, respectively. Temperature-dependent I-V data were collected in the high-temperature regime (above room temperature) to avoid weak conductivity variations (see the Supporting Information, Figure S10). Such variations occur because at low temperature in the off state, the carriers do not have sufficient energy to pass through the barrier, and thermionic emission theory fails to explain this behavior (a detailed discussion can be found in ref. 29). To determine the SBH, the reciprocal temperature dependence of the ln(I_DS_/T^2^) fit curve was plotted for various gate voltages at the Au/MoS_2_ (Fig. 3b) and MGr/MoS_2_ (Fig. 3c) interfaces. The slope, S, was extracted from the different V_BG_ values of the Arrhenius plot, and the intercept, S_INT_, yielded the Schottky barrier height, Φ_SB_, as a function of the gate voltage, V_BG_, using the expression S_INT_ = −eΦ_SB_/1000 k_B_ (see the Supporting Information, Figure S11). For the MGr/MoS_2_ devices, the slope of the linear fit curve in the Arrhenius plot is negative near the off state (low V_BG_; −5 V) and becomes more positive with the formation of a highly conductive MoS_2_ channel at V_BG_ = −1.6 V, as depicted in Fig. 3d. The resulting features imply that the SBH dramatically decreases from 300 to 0 meV, becomes negative, and finally saturates near −45.5 meV, as shown in Fig. 3e. This finding represents the first observation of negative SBH behavior at an MGr/MoS_2_ contact and is contrary to the results of several studies that have investigated this phenomenon in the 0–300 meV range^16^,^17^,^18^,^19^. Similar findings have been reported in conventional semiconductors and novel 2D materials, *e.g.*, MoS_2_ with ferromagnetic permalloy contacts^29^, p-type MoS_2_ with MoO_X_^30^, and silicon (001) with metal Ti^31^. By contrast, for the Au/MoS_2_ contact considered in this study, under a negative gate bias, the current through the Schottky barrier *via* thermionic emission resulted in a linearly decreasing trend (from 765.9 to 118.8 meV) as the gate bias became forward directed.

### Negative Schottky barrier behavior

Generally, the semiconductor surface states and the work functions of the metal play a decisive role in the determination of the SBH, but the details of Schottky barrier formation in metal/semiconductor systems are not well understood. Various previous studies on metal/MoS_2_ contacts have demonstrated an independence of the metal work functions due to the presence of interfacial states, D_IT_, that cause Fermi-level pinning^13^,^14^,^32^,^33^. By combining the experimental data presented in Figure S12 of the Supporting Information, we estimated D_IT_ using the expression^28^ D_IT_ ≈ 1.1 × 10^13^(1 − S_0_)/S_0_ ≈ 7.9 × 10^13^ states/eV cm^2^, where the slope is S_0_ = dΦ_SB_/dΦ_M_ ≈ 0.12 and Φ_M_ is the metal work function. However, when MGr and MoS_2_ are brought into contact, the Schottky-Mott model does not hold, because D_IT_ ≠ 0. A modified model can be written as follows^34^:


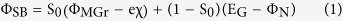


where Φ_MGr_ = 4.5 eV and χ = 4.0 eV are the work function of the graphene and the electron affinity of the MoS_2_^35^,^36^, respectively; E_G_ = 1.6 eV is the band gap of the bi-layer MoS_2_; and Φ_N_ = 1.53 eV is the neutral level of the interface states. In practice, the Fermi level is pinned at a certain energy, and then, a depletion contact with a positive Schottky barrier is created at the MGr/MoS_2_ interface. Considering this behavior, the first term of equation (1) will change to a negative value under the influence of the gate voltage, forming an accumulation contact (with a negative barrier height), at which the preferred ohmic contact can be formed (as shown in Fig. 3f). Because Φ_MGR_ depends on the back-gate electric field, the carrier density in the MGr shifts the Fermi level by ΔE_F,MGr_ = ħν_F_√νr|C_OX_/e(V_BG_ − V_T_)|), where ν_F_ is the Fermi velocity. The tunability of the work function of graphene has been demonstrated by Yu, Y.-J. *et al.*^37^ only for the single- and bi-layer cases, not for thick layers such as those used in our experiments. Furthermore, if the work function of the multi-layered graphene, Φ_MGr_, is less than that of the MoS_2_, *i.e.,* Φ_B_(V_BG_) = Φ_MGr_ − Φ_MoS2_(V_BG_), then it is possible for a negative contact potential, Φ_B_(V_BG_), to arise as shown in Fig. 3f. As demonstrated by Li, Y. *et al.*^38^, the electric-field-driven modulation of the work function of MoS_2_ is estimated to be Φ_MoS2_(V_BG_) = e(χ + ϕ_S_) − ħπ(C_OX_/e)(V_BG_ − V_T_)/2 m^*^, where ϕ_S_, ħ, and m^*^ are the surface potential, Planck’s constant, and the effective mass of bi-layer MoS_2_, respectively. For the values given in ref. 38, the Fermi-level variation, ΔE_F,MoS2_, of the bi-layer at the turning point voltage (V_BG _= −3.8 V) is only approximately 31 meV, which is quite small. Therefore, we suggest that the tunable Schottky barrier is primarily responsible for the modulation of the work function of the thick graphene. Despite the large number of graphene layers, ohmic contacts can be formed, which will provide new opportunities for the engineering of highly efficient contacts in flexible electronics and photonics applications.

## Discussion

In summary, we fabricated heterostructured MoS_2_ FETs with multi-layered graphene contacts and systematically investigated their electronic properties at the MGr/MoS_2_ interface. Negligible hysteresis was observed because of the optimized fabrication procedure. Upon the insertion of an intermediate tunnel layer, the devices displayed highly improved performance in terms of carrier mobility. An interesting negative Schottky barrier behavior was observed between the MoS_2_ and the multi-layered graphene. We also qualitatively discussed the formation of ohmic contacts, which likely contributed to the controllability of the graphene work function by gate bias modulation. Thus, we were able to improve the carrier injection behavior and reduce the contact resistance, thereby developing a new possible approach for future electronics applications.

## Methods

Multi-layered graphene was mechanically exfoliated from single-crystalline graphite (from Graphene Supermarket) using the standard Scotch-tape-based cleavage method. The thick graphene flakes were transferred onto bottom gold electrodes, which served as the source and drain, using techniques described previously^39^. In brief, this technique was based on the use of a viscoelastic PDMS layer that served as an acceptor surface in place of a silicon substrate. The 2D flakes were deposited on the PDMS layer and then aligned on top of the Au pads using optical microscopy. We then brought the 2D/PDMS stack into contact with the target substrate and slowly peeled it away from the PDMS.

Before the dry transfer was performed, the gold electrodes were pre-patterned on a degenerately doped Si substrate covered with a 280-nm layer of SiO_2_. We intentionally chose two similar thicknesses of graphene flakes to perform the above techniques twice. Subsequently, MoS_2_ (2D semiconductor) flakes were precisely deposited on top of the graphene contacts to serve as a channel material. The fabrication process described above avoids the use of any chemical solutions to minimize contamination. The devices were subsequently annealed at 399 K for 12 hours in a cryostat (ASK, 700 K) at a base pressure of ~2 × 10^−2^ Torr for the removal of adsorbates. Electrical measurements were performed using a semiconductor parameter analyzer (HP, 4156A) after *in situ* vacuum annealing. C-V measurements were performed using a Precision LCR meter (Agilent, E4980A) with the samples in the same chamber.

A focused ion beam (FEI, Quanta 3D FEG) was employed to prepare cross-sectional TEM samples after the deposition of a carbon/Pt coating. HRTEM imaging was performed using a spherical-aberration-corrected TEM (JEOL, JEM-2100F) operating at 200 kV. Chemical composition mapping was performed with an EDX apparatus attached to the TEM instrument.

The numbers of layers of graphene and MoS_2_ were determined based on the color contrast in the optical microscope images and were further confirmed by AFM (Park Systems, XE-100). Raman spectroscopy (Jaco, NRS-3100) with laser excitation at λ = 532 nm was employed to analyze the spectra of the MoS_2_ reference flakes and the FET channels.

## Additional Information

**How to cite this article**: Qiu, D. and Kim, E. K. Electrically Tunable and Negative Schottky Barriers in Multi-layered Graphene/MoS_2_ Heterostructured Transistors. *Sci. Rep.* **5**, 13743; doi: 10.1038/srep13743 (2015).

## Supplementary Material

Supplementary Information
