# Supplementary material for: Electrically Tunable and Negative Schottky Barriers in Multi-layered Graphene/MoS2 Heterostructured Transistors
Source: Sci Rep. 2015 Sep 3;5:13743. doi: 10.1038/srep13743 (PMC4558713; doi:10.1038/srep13743)
Supplement: Supplementary Information [file srep13743-s1.doc]

**Supplementary Information for**

**Electrically Tunable and Negative Schottky Barriers in Multi-layered Graphene/MoS_2_ Heterostructured Transistors**

Dongri Qiu and Eun Kyu Kim^*^

*Quantum-Function Research Laboratory and Department of Physics, Hanyang University,*

*Seoul 133-791, South Korea*

^*^To whom correspondence should be addressed: ek-kim@hanyang.ac.kr

**Dry transfer method using PDMS**


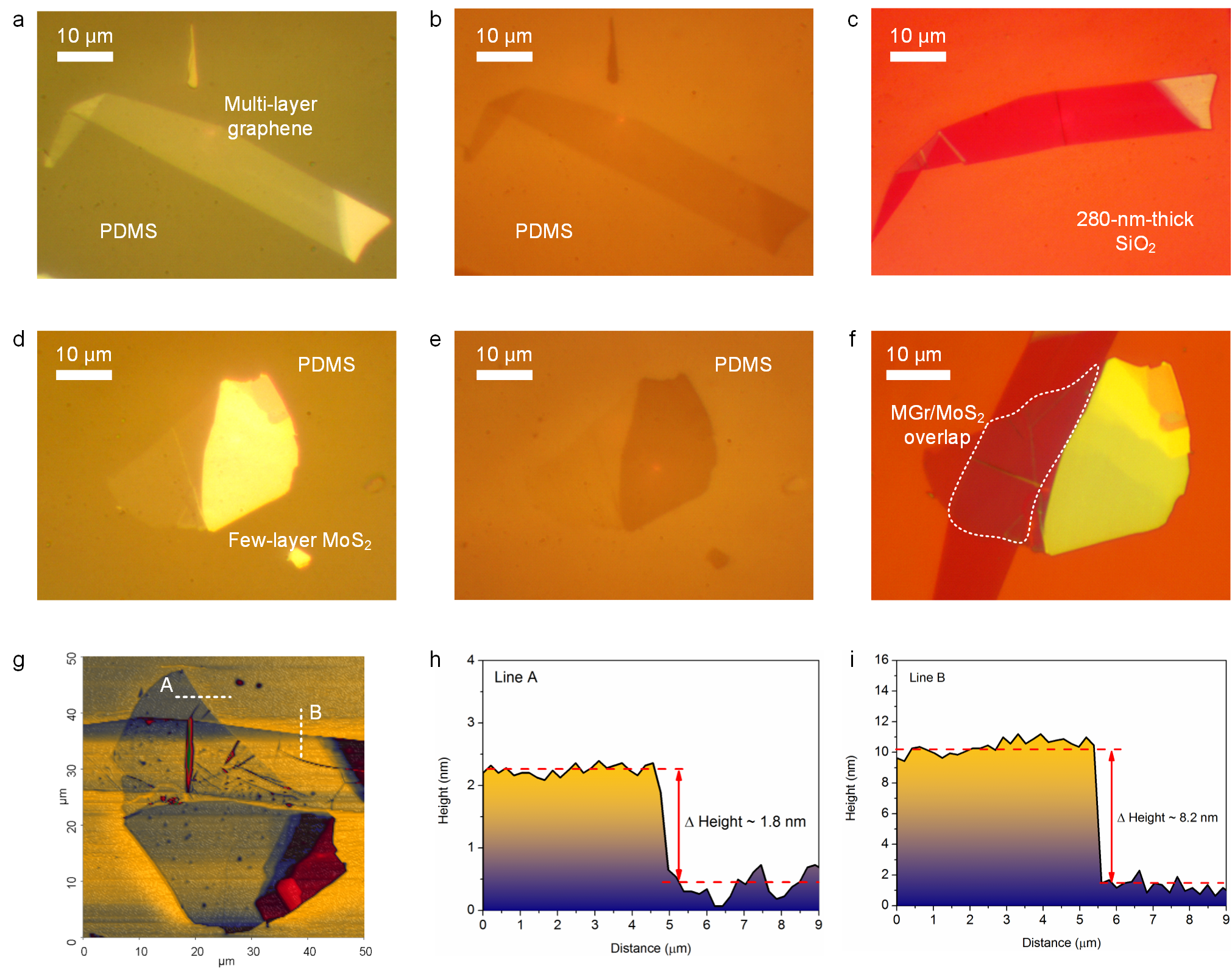


**Figure S1.** Optical images of exfoliated multi-layered graphene on a PDMS layer acquired in (a) backscattering and (b) transmission modes. (c)-(e) Optical microscopy images of (c) MGr transferred onto a silicon substrate and of few-layered MoS_2_ acquired in (d) backscattering and (e) transmission modes. (f) MoS_2_ flakes transferred onto the MGr to create a heterostructure. (g) The corresponding AFM image of the overlapped 2D materials. (h)-(i) Cross-sectional profiles along lines A and B in (g).


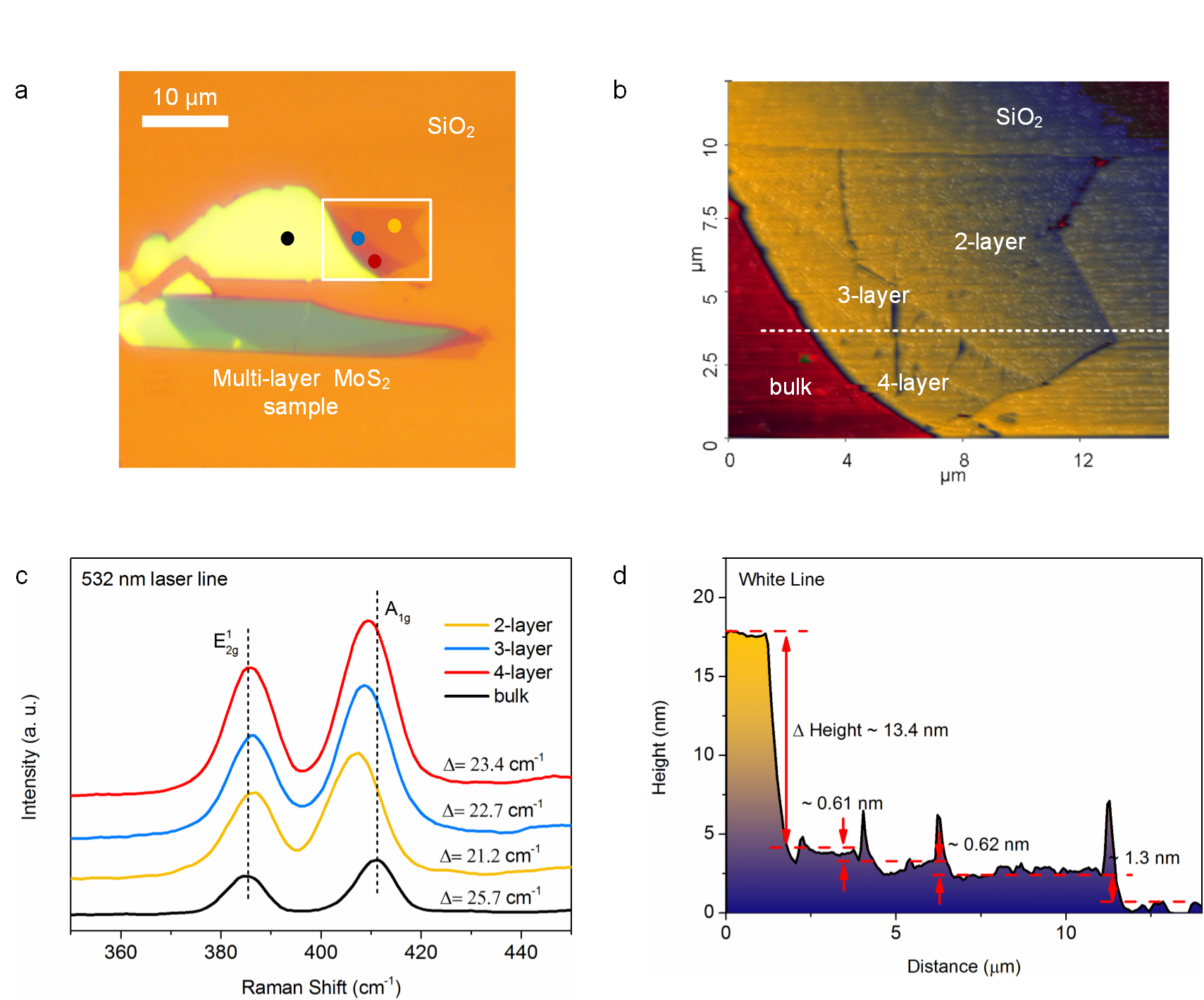
**Material characterization of the mechanically exfoliated MoS_2_ flakes**

**Figure S2.** (a) Optical micrograph of a multi-layered MoS_2_ sample. (b) AFM image of the area inside the white box in (a). (c) Raman signals acquired from the different locations highlighted by the colored dots in (a), corresponding to different layer thicknesses of MoS_2_. The Raman peak difference (Δ) between E^1^_2g_ and A_1g_ ranges from 22.2 cm^-1^ for the bi-layer to 25.7 cm^-1^ for the bulk. (d) AFM height profile along the white line in (b).


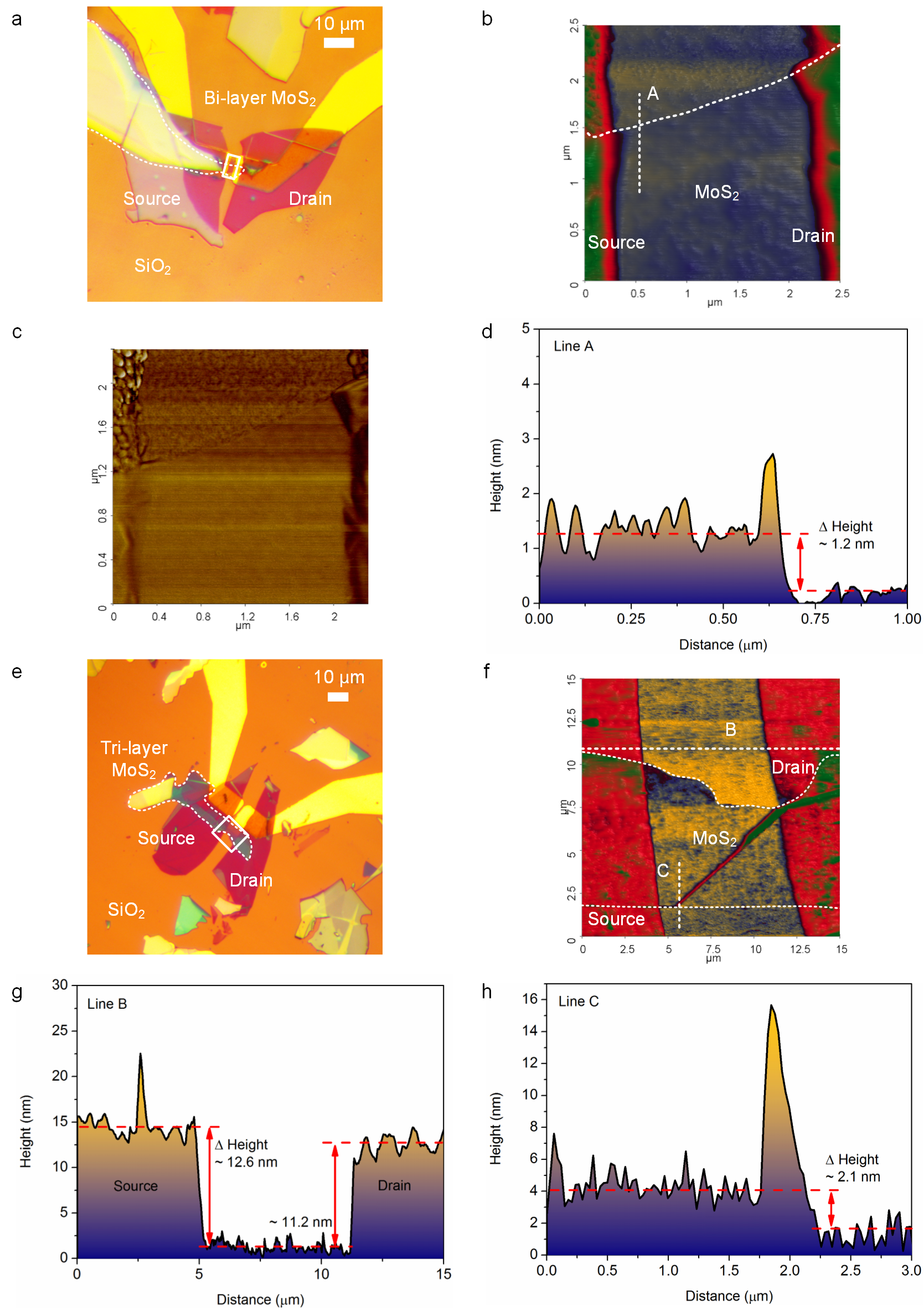
**AFM characterization of the MGr/MoS_2_ devices**

**Figure S3.** (a) A magnified optical image of an MGr/MoS_2_ FET, as described in the manuscript (shown in Figure 1b). (b) AFM topography and (c) phase image of the selected region of the FET shown in (a). (d) Height profile along line A indicated in the AFM image. (e) Optical image of a tri-layer MoS_2_ FET with graphite contacts. (f) AFM image. (g)-(h) The height profiles along the white lines labeled (g) B and (h) C in (f).


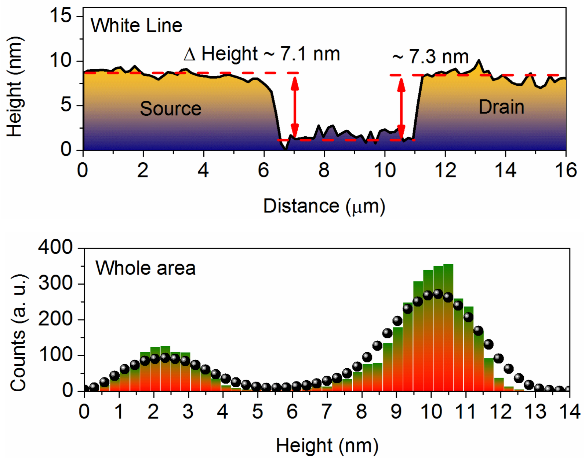
**Height and statistical studies based on the AFM measurements**

**Figure S4.** Top: The AFM cross-sectional profile along the white line in Figure 1c in the manuscript. Bottom: Distribution histogram recorded for the total area of the AFM scan (16×16 μm^2^).

**Elemental composition spectra obtained using EDX**

**
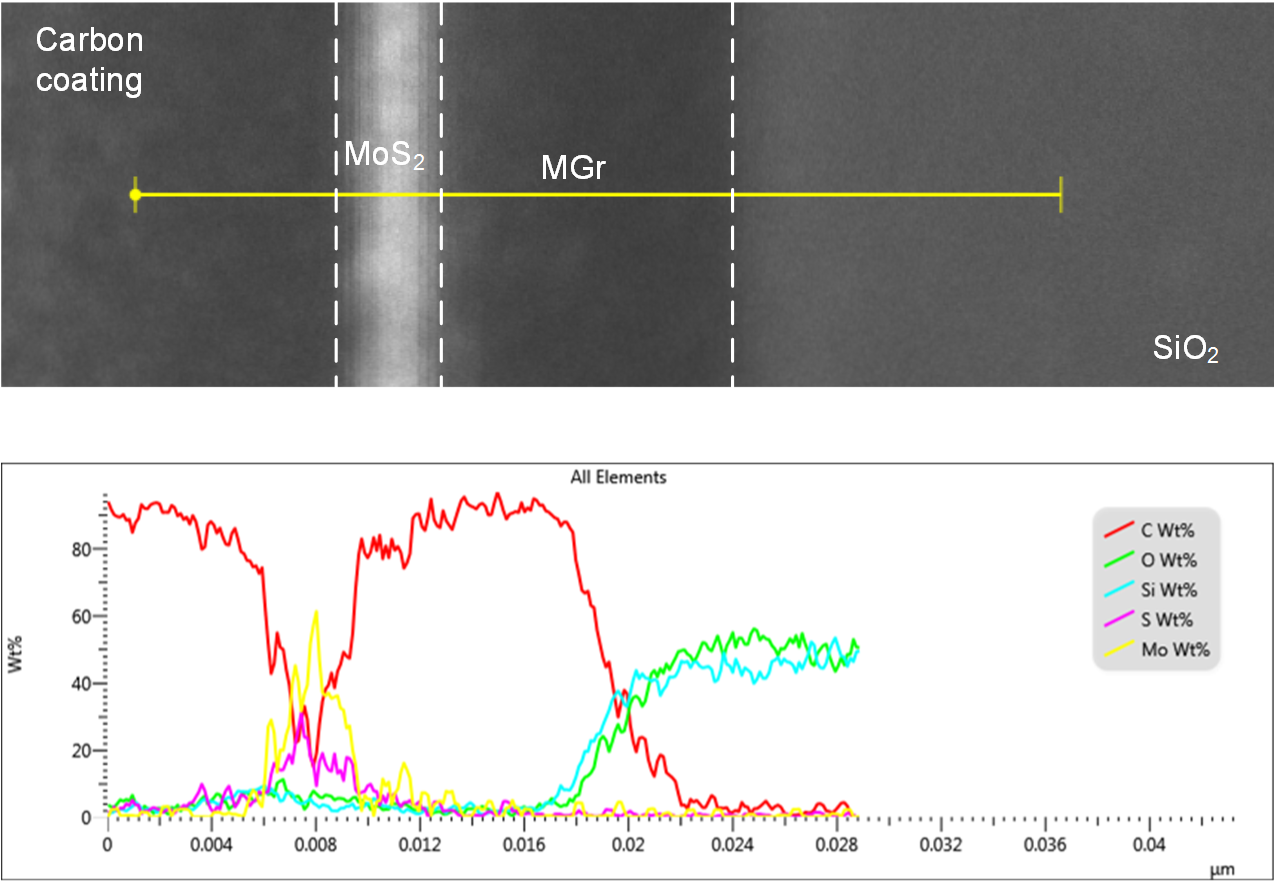
**

**Figure S5.** Top: STEM image of the MGr/MoS_2_ heterostructure. Bottom: The spectra for the C, O, Si, S, and Mo elements.


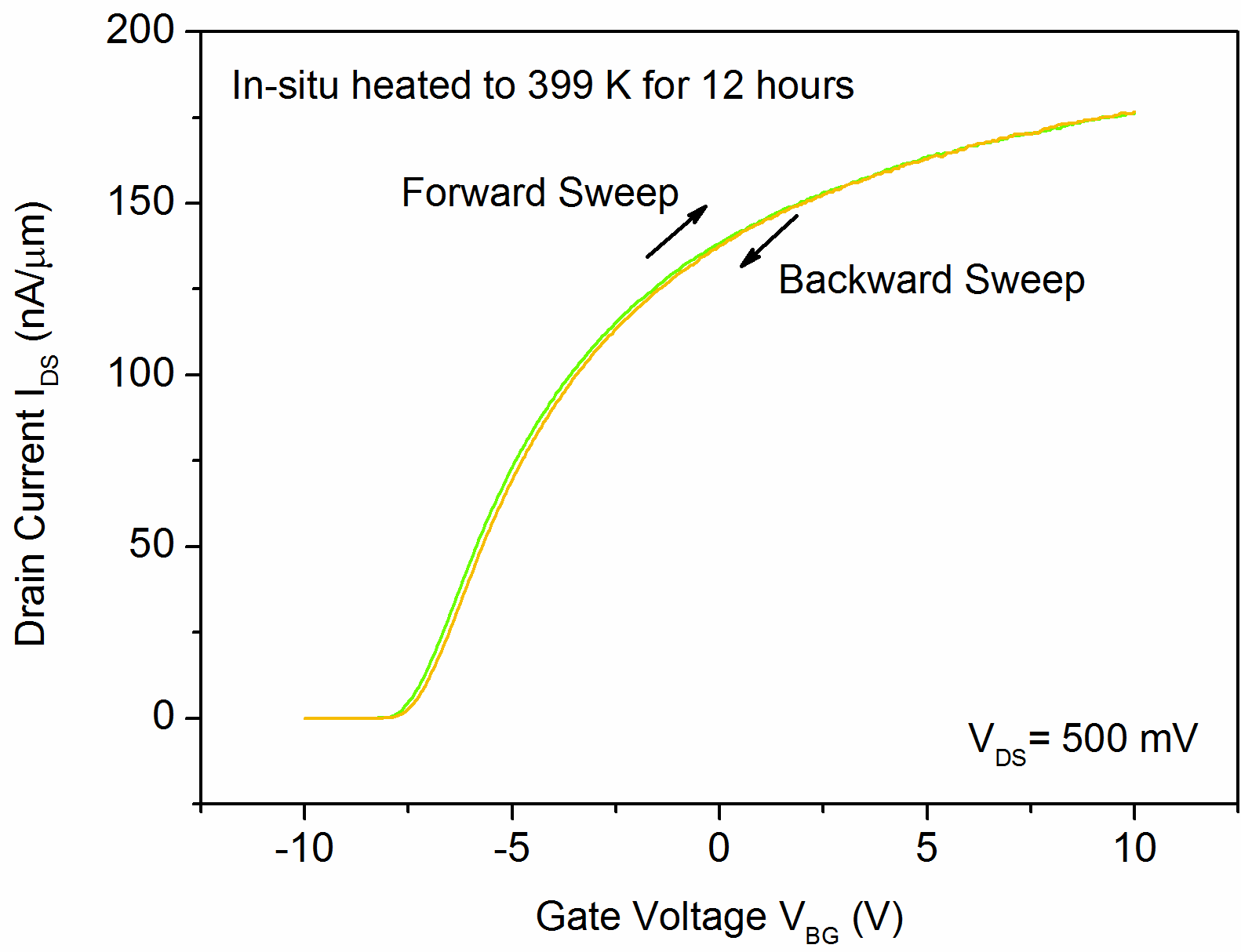
**I-V hysteresis behavior**

**Figure S6.** The hysteretic characteristics of I_DS_-V_BG_ sweeps from -10 to 10 V (forward sweep) and from 10 to -10 V (backward sweep) at V_DS_=500 mV. We observed negligible hysteresis after applying an *in situ* annealing process (at 399 K for over 12 hours at a pressure of ~ 2×10^-2^ Torr).

**Characterization of the Au/MoS_2_ devices**

**
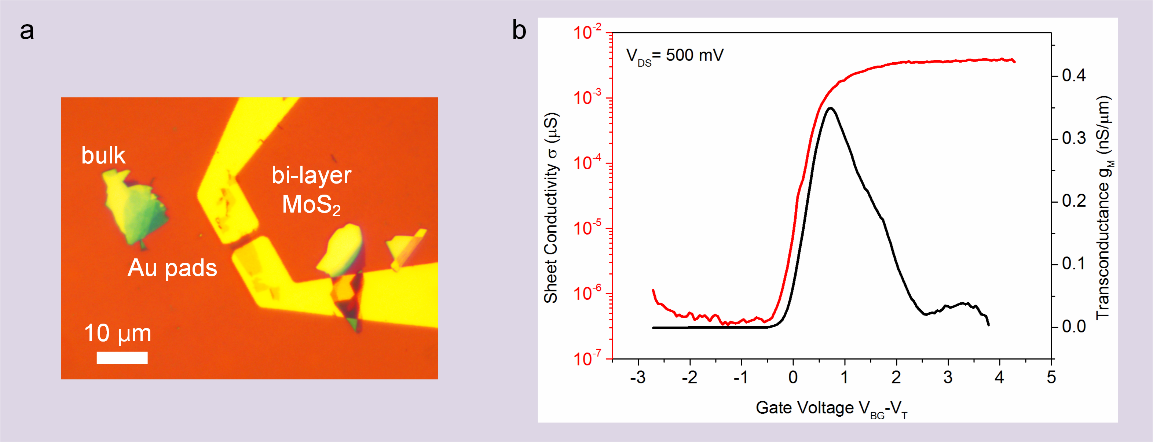
**

**Figure S7.** (a) Optical image of a bi-layer MoS_2_ flake deposited on Au pads. (b) Electrical transport characteristics of the bi-layer MoS_2_ FET.


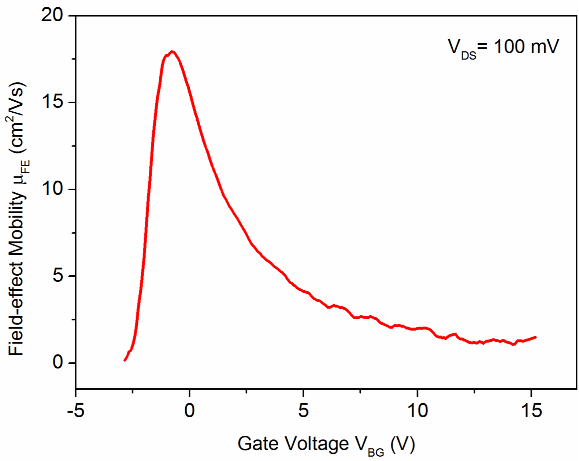
**Field-effect mobility extraction**

**Figure S8.** The extracted field-effect mobility as a function of the gate voltage at room temperature.


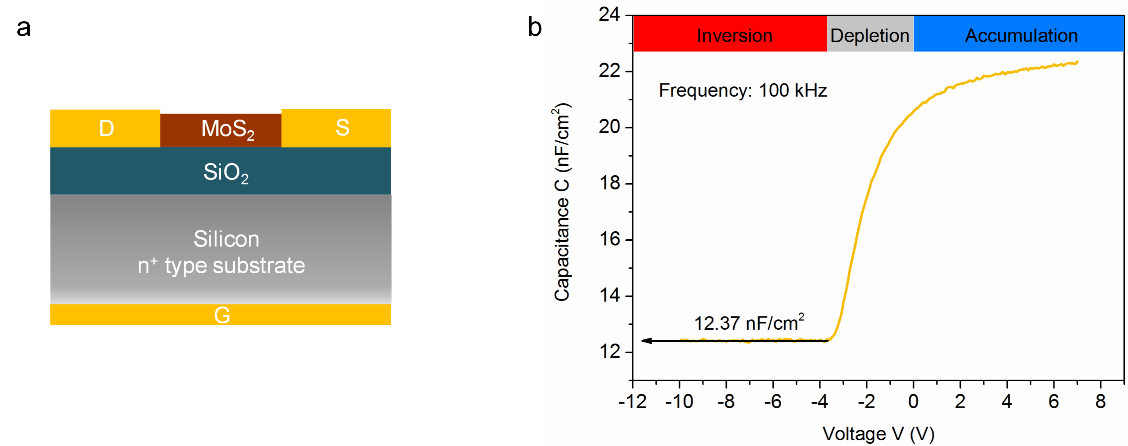
**C-V measurements**

**Figure S9.** (a) Schematic representation of the actual device configuration. (b) Measured C-V curve of the capacitor at 100 kHz.


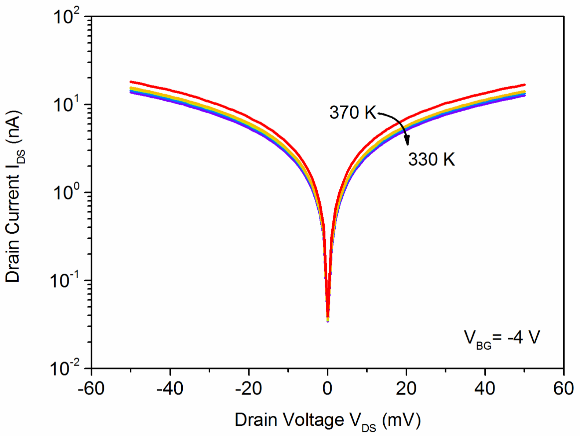
**I-V output curves**

**Figure S10.** Temperature-dependent semi-log I_DS_-V_DS_ output curves of a bi-layer MoS_2_ FET with an MGr contact.

**SBH extraction**

**Figure S11.** (a) Arrhenius plot of ln(I_DS_/T^2^)-1000/T for various values of V_DS_ for the Au/MoS_2_ system at V_BG_= -2 V and (b) the corresponding drain voltage dependence of the slope S for SBH extraction. (c) The same Arrhenius plot for the MGr/MoS_2_ system and (d) the drain bias dependence of the slope S for this system.


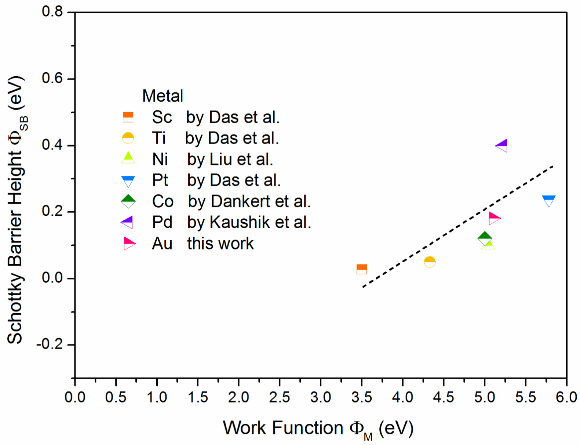
**Analysis of the metal-to-MoS_2_ interface properties**

**Figure S12.** Schottky barrier heights for various metals. The experimental data were obtained from previous published studies and our work.^1-4^ The dashed line is the fit result, which yields a slope of S_0_=0.12. Note that the slope S_0_ should to be unit at ideal surface states in the metal-to-semiconductor system.

**References**

S1 Das, S., Chen, H. Y., Penumatcha, A. V. & Appenzeller, J. High Performance Multilayer MoS_2_ Transistors with Scandium Contacts. *Nano Lett.* **13**, 100-105, (2013).

S2 Liu, H., Neal, A. T. & Ye, P. D. Channel length scaling of MoS_2_ MOSFETs. *ACS Nano* **6**, 8563-8569 (2012).

S3 Dankert, A., Langouche, L., Kamalakar, M. V. & Dash, S. P. High-Performance Molybdenum Disulfide Field-Effect Transistors with Spin Tunnel Contacts. *ACS Nano* **8**, 476-482 (2014).

S4 Kaushik, N. *et al.* Schottky barrier heights for Au and Pd contacts to MoS_2_. *Appl. Phys. Lett.* **105**, 113505 (2014).
